# CORRIGENDUM

**DOI:** 10.48101/ujms.v130.12186

**Published:** 2025-04-03

correction for ”Cost-effectiveness analysis of transcatheter aortic valve implantation versus surgical aortic valve replacement in patients with severe aortic stenosis at low risk of surgical mortality in Sweden” Ups J Med Sci. 2024;129: e10741. doi: 10.48101/ujms.v129.10741

This corrigendum is published as the authors have discovered a minor mistake in the model used in the publication. The ‘alive and well’ health state costs in the model were wrongly accounted for as ‘yearly’ instead of ‘monthly’. However, accounting for this change does not impact the main message of the study - there is in fact a lower resultant ICER value, implying that the results are even more positive for TAVI.

The table below highlights the main changes reflected in the published, updated version - values for the ‘Alive and well’ health state cost and the resultant ICER:

**Table:** 

	**Value in original published version** (Yearly cost instead of monthly)	**Corrected value in updated version** (Monthly cost)
‘Alive and Well’ Health state cost- TAVI and SAVR- Year 1	8215 SEK	685 SEK
‘Alive and Well’ Health state cost- TAVI and SAVR- Year 2 onwards	4108 SEK	342 SEK
Incremental cost effectiveness ratio (ICER)	343,918 SEK	76,532 SEK/QALY

The online version of this paper has been corrected to reflect these changes. The authors would like to apologize for the mistake.

