# Supplementary material for: Cost-effectiveness analysis of transcatheter aortic valve implantation versus surgical aortic valve replacement in patients with severe aortic stenosis at low risk of surgical mortality in Sweden
Source: Ups J Med Sci. 2024 Nov 7;129:10.48101/ujms.v129.10741. doi: 10.48101/ujms.v129.10741 (PMC11650470; doi:10.48101/ujms.v129.10741)
Supplement: Supplementary file 1 [file UJMS-129-10741-s1.pdf]

Supplementary material

Table S1. Probabilities of clinical events used in the model.

Clinical events	TAVI with SAPIEN 3	SAVR	Source
**At 30 days**			
Treated AF	0.98%	21.24%	SWEDEHEART registry (2018-2020)[6]
New permanent pacemaker	1.47%	0.00%	
Rehospitalisation	13.73%	58.11%	
Disabling stroke	1.47%	1.53%	
Aortic reintervention	0.00%	0.07%	
Mortality	0.98%	1.45%	
**Monthly health states transition probabilities and intercurrent events between 30 days and 1 year**			
Alive and well → Treated AF	0.23%	0.13%	SWEDEHEART registry (2018-2020)[6]
Alive and well → Disabling stroke	0.04%	0.01%	
Treated AF → Disabling stroke	0.04%	0.01%	
TIA	0.00%	0.01%	
MI	0.00%	0.00%	
Severe or life-threatening bleeding	0.00%	0.12%	SWEDEHEART registry (2018-2020)[6] PARTNER 3[9]
**Monthly health states transition probabilities after 1 year and beyond**			
Alive and well → Treated AF	0.00%	0.00%	SWEDEHEART registry (2018-2020)[6]
Alive and well → Disabling stroke	0.00%	0.00%	
Treated AF → Disabling stroke	0.00%	0.00%	
**Events beyond 30 days (converted to monthly rates in calculations)**			
Rehospitalisation for first 12 months	43.94%	10.68%	SWEDEHEART registry (2018-2020)[6]
Rehospitalisation from month 13 to month 24	6.30%	4.70%	

Rehospitalisation from month 25 to month 36	12.20%	2.90%	
Rehospitalisation from month 37 onwards	0.00%	0.00%	
**Aortic reintervention** (converted to monthly rates in calculations)			
Annual probability	0.07%	0.07%	SWEDEHEART registry (2018-2020)[6]

AF, atrial fibrillation; TIA, transient ischaemic stroke; MI, myocardial infarction; TAVI, transcatheter aortic valve implantation.

Table S2. Annual mortality risk for 'alive and well' and associated relative risk for other health states.

Annual mortality risk - alive and well (by age)	Males	Females	Monthly probability for TAVI	Monthly probability for SAVR	Source
70 years	1.57%	1.05%	0.11%	0.11%	National Life Tables Sweden (2022)[27]
71 years	1.67%	1.11%	0.12%	0.12%	
72 years	2.01%	1.34%	0.14%	0.14%	
73 years	2.12%	1.50%	0.15%	0.15%	
74 years	2.36%	1.71%	0.17%	0.17%	
75 years	2.75%	1.81%	0.19%	0.19%	
76 years	2.98%	2.01%	0.21%	0.21%	
77 years	3.26%	2.32%	0.24%	0.24%	
78 years	3.56%	2.53%	0.26%	0.26%	
79 years	4.33%	2.92%	0.31%	0.31%	
80 years	4.79%	3.36%	0.35%	0.35%	
81 years	5.32%	3.66%	0.39%	0.39%	
82 years	6.18%	4.47%	0.46%	0.46%	
83 years	6.97%	5.11%	0.52%	0.52%	
84 years	7.81%	5.69%	0.58%	0.58%	
85 years	9.05%	6.47%	0.68%	0.68%	
86 years	10.48%	7.67%	0.80%	0.80%	
87 years	11.51%	8.71%	0.89%	0.89%	
88 years	13.14%	9.96%	1.02%	1.02%	
89 years	14.62%	10.91%	1.14%	1.14%	
90 years	17.16%	12.84%	1.35%	1.35%	
91 years	18.00%	14.19%	1.46%	1.46%	
92 years	20.92%	16.73%	1.73%	1.73%	
93 years	23.43%	18.16%	1.94%	1.94%	

94 years	25.20%	19.90%	2.12%	2.12%	
95 years	27.27%	22.08%	2.35%	2.35%	
96 years	29.74%	24.30%	2.60%	2.60%	
97 years	32.36%	26.68%	2.89%	2.89%	
98 years	35.12%	29.23%	3.20%	3.20%	
99 years	38.02%	31.94%	3.54%	3.54%	
100 years	41.04%	34.82%	3.91%	3.91%	
**Relative risk of death (HR) associated with treated AF, disabling stroke and reintervention**					
Treated AF	HR=1.46	Odutayo et al.[28]			
Disabling stroke	HR=2.30	Dennis et al.[29]			
Aortic reintervention	HR=2.21	PARTNER 3 trial[9]<br>Procedural deaths (0.4%) compared to monthly mortality risk of general mortality for 73 years old[a] (0.16%)			

[a] Average age of the PARTNER 3 cohort

AF, atrial fibrillation; HR, hazard ratio; SAVR, surgical aortic valve replacement; TAVI, transcatheter aortic valve implantation.

Table S3. Deterministic sensitivity analysis input parameters.

**Parameter**	**Base case value**	**Lower value**	**Upper value**	**Lower (%)**	**Upper (%)**
Age, years	76	65	85	14.5%	11.8%
Proportion male	52%	41.8%	62.6%	20.0%	20.0%
Discount rate: costs (1–30 years)	3.0%	2.4%	3.6%	20.0%	20.0%
Discount rate: costs (after 30 years)	3.0%	2.4%	3.6%	20.0%	20.0%
Discount rate: benefits (1–30 years)	3.0%	2.4%	3.6%	20.0%	20.0%
Discount rate: benefits (after 30 years)	3.0%	2.4%	3.6%	20.0%	20.0%
TAVI with SAPIEN 3: Mortality risk at 30 days	1.0%	0.8%	1.2%	20.0%	20.0%
TAVI with SAPIEN 3: Risk of new onset of treated AF at 30 days	1.0%	0.8%	1.2%	20.0%	20.0%
TAVI with SAPIEN 3: Risk of new PP at 30 days	1.5%	1.2%	1.8%	20.0%	20.0%
TAVI with SAPIEN 3: Risk of DS at 30 days	1.5%	1.2%	1.8%	20.0%	20.0%
SAVR: Mortality risk at 30 days	1.5%	1.2%	1.8%	20.0%	20.0%
SAVR: Risk of new onset treated AF at 30 days	21.2%	16.9%	25.5%	20.0%	20.0%
SAVR: Risk of new PP at 30 days	0.0%	0.0%	0.0%	NA	NA
SAVR: Risk of DS at 30 days	1.5%	1.2%	1.8%	20.0%	20.0%
TAVI with SAPIEN 3: Alive and well to treated AF (monthly transition after 1 year)	0.0%	0.0%	0.0%	NA	NA

TAVI w. SAPIEN 3: Alive and well to DS (monthly transition after 1 year)	0.0%	0.0%	0.0%	NA	NA
TAVI w. SAPIEN 3: Treated AF to DS (monthly transition after 1 year)	0.0%	0.0%	0.0%	NA	NA
SAVR: Alive and well to treated AF (monthly transition after 1 year)	0.0%	0.0%	0.0%	NA	NA
SAVR: Alive and well to DS (monthly transition after 1 year)	0.0%	0.0%	0.0%	NA	NA
SAVR: Treated AF to DS (monthly transition after 1 year)	0.0%	0.0%	0.0%	NA	NA
TAVI with SAPIEN 3 : Rehospitalisation multiplier	1	0.8	1.2	20.0%	20.0%
SAVR: Rehospitalisation multiplier	1	0.8	1.2	20.0%	20.0%
TAVI with SAPIEN 3 : Reintervention multiplier	1	0.8	1.2	20.0%	20.0%
SAVR: Reintervention multiplier	1	0.8	1.2	20.0%	20.0%
RR of death for treated AF	1.5	1.2	1.8	4.8%	5.5%
RR of death with DS (month 1)	2.3	1.8	2.7	20.0%	20.0%
RR of death with DS (month 2+)	2.3	1.8	2.7	20.0%	20.0%
RR of death with reintervention TAVI with SAPIEN 3	2.2	1.8	2.7	20.0%	20.0%
RR of death with reintervention SAVR	2.2	1.8	2.7	20.0%	20.0%
HR mortality vs SAVR: TAVI with SAPIEN 3	0.8	0.4	1.6	53.3%	117.3%
Utility decrement: treated AF	0.08	0.07	0.10	20.0%	20.0%
Utility decrement: DS	0.36	0.29	0.44	20.0%	20.0%

Procedure cost: TAVI with SAPIEN 3	302 329 SEK	241 863 SEK	362 795 SEK	20.0%	20.0%
Procedure cost: SAVR	244 750 SEK	195 800 SEK	293 700 SEK	20.0%	20.0%
AEs cost: TAVI with SAPIEN 3	0	0	311	NA	NA
AEs cost: SAVR	0	0	1 281	NA	NA
AF cost: per month from month 2	89	71	66 673	20.0%	20.0%
DS cost (month 1): per month	8 235	6 588	2 533	20.0%	20.0%
DS cost (month 2+): per month	3 786	3 029	210 787	20.0%	20.0%
Alive and well cost: per month (SAVR 2+)	38	30	50 705	20.0%	20.0%
PP insertion cost	7 841	6 273	9 858	20.0%	20.0%
PP complications (per month)	56	44	4 930	20.0%	20.0%
Hospitalisation cost TAVI with SAPIEN 3	3 093	2 474	9 858	20.0%	20.0%
Hospitalisation costs SAVR	3 093	2 474	4 930	20.0%	20.0%
Reintervention cost with TAVI with SAPIEN 3	302 329	241 863	362 795	20.0%	20.0%
Reintervention cost with SAVR	302 329	241 863	362 795	20.0%	20.0%

AE, adverse events; AF, atrial fibrillation; DS, disabling stroke; HR, hazard ratio; PP, permanent pacemaker; RR, relative risk; SAVR, surgical aortic valve replacement; TAVI, transcatheter aortic valve implantation.

Table S4. Probabilistic sensitivity analysis assumptions.

Parameter	Base case value	Distribution	Parameters	Source
**TAVI with SAPIEN 3 – clinical events at 30 days**				
All-cause mortality	0.98%	Beta	Alpha = 2 / Beta = 202	SWEDEHEART registry (2018-2020)[6]
New onset of Treated AF	0.98%	Beta	Alpha = 2 / Beta = 202	
New permanent pacemaker	1.47%	Beta	Alpha = 3 / Beta = 201	
Rehospitalisation	13.73%	Beta	Alpha = 28 / Beta = 176	
Disabling stroke	1.47%	Beta	Alpha = 3 / Beta = 201	
Non-disabling stroke	0.60%	Beta	Alpha = 3 / Beta = 493	PARTNER 3 trial[9]
TIA	0.00%	Beta	Alpha = 0 / Beta = 204	SWEDEHEART registry (2018-2020)[6]
MI	0.00%	Beta	Alpha = 0 / Beta = 204	
Bleeding	4.90%	Beta	Alpha = 10 / Beta = 194	
AKI with renal replacement therapy	0.00%	Beta	Alpha = 0 / Beta = 204	
Aortic reintervention	0.00%	Beta	Alpha = 0 / Beta = 204	
**SAVR – clinical events at 30 days**				
All-cause mortality	1.45%	Beta	Alpha = 20 / Beta = 1355	SWEDEHEART registry (2018-2020)[6]
New onset of Treated AF	21.24%	Beta	Alpha = 292 / Beta = 1083	
New permanent pacemaker	0.00%	Beta	Alpha = 0 / Beta = 1375	

Rehospitalisation	58.11%	Beta	Alpha = 733 / Beta = 576	
Disabling stroke	1.53%	Beta	Alpha = 21 / Beta = 1354	
Non-disabling stroke	1.98%	Beta	Alpha = 9 / Beta = 445	PARTNER 3 trial[9]
TIA	0.07%	Beta	Alpha = 1 / Beta = 1374	SWEDEHEART registry (2018-2020)[6]
MI	1.24%	Beta	Alpha = 17 / Beta = 1358	
Bleeding	24.45%	Beta	Alpha = 111 / Beta = 343	PARTNER 3 trial[9]
AKI with renal replacement therapy	0.00%	Beta	Alpha = 0 / Beta = 1375	SWEDEHEART registry (2018-2020)[6]
Aortic reintervention	0.07%	Beta	Alpha = 1 / Beta = 1374	
**TAVI with SAPIEN 3 – Clinical events from 30 days to 1 year (monthly probability)**				
TIA	0.00%	Beta	Alpha = 0 / Beta = 204	SWEDEHEART registry (2018-2020)[6]
MI	0.00%	Beta	Alpha = 0 / Beta = 204	
Severe/life-threatening bleeding	0.00%	Beta	Alpha = 0 / Beta = 204	
Rehospitalisation rate	43.94 %	Gamma	SD = 0.04 (assumption)	
**SAVR – Clinical events from 30 days to 1 year (monthly probability)**				
TIA	0.01%	Beta	Alpha = 1 / Beta = 1374	SWEDEHEART registry (2018-2020)[6]
MI	0.00%	Beta	Alpha = 0 / Beta = 1375	
Severe/life-threatening bleeding	0.12%	Beta	Alpha = 6 / Beta = 448	PARTNER 3 trial[9]

Rehospitalisation rate	10.68 **%**	Gamma	SD = 0.01% (assumption)	SWEDEHEART registry (2018-2020)[6]
**TAVI with SAPIEN 3 – Monthly transition probabilities (Month 1 to 12 and Month 13 onwards)**				
Alive and well to Treated AF – Month 1 to Month 12	0.23%	Beta	Alpha = 5 / Beta = 199	SWEDEHEART registry (2018-2020)[6]
Alive and well to Treated AF – Month 13 onwards	0.00%	Beta	Alpha = 0 / Beta = 204	
Alive and well to Disabling stroke– Month 1 to Month 12	0.041%	Beta	Alpha = 1/ Beta = 203	
Alive and well to Disabling stroke– Month 13 onwards	0.00%	Beta	Alpha = 0 / Beta = 204	
Treated AF to Disabling stroke– Month 1 to Month 12	0.04%	Beta	Alpha = 55 / Beta = 587.812	
Treated AF to Disabling stroke– Month 13 onwards	0.00%	Beta	Alpha = 0 / Beta = 204	
**SAVR – Monthly transition probabilities (Month 1 to 12 and Month 13 onwards)**				
Alive and well to Treated AF – Month 1 to Month 12	0.13%	Beta	Alpha = 19 / Beta = 1356	SWEDEHEART registry (2018-2020)[6]
Alive and well to Treated AF – Month 13 Onwards	0.00%	Beta	Alpha = 0 / Beta = 1375	
Alive and well to Disabling stroke– Month 1 to Month 12	0.006%	Beta	Alpha = 1 / Beta = 1374	

Alive and well to Disabling stroke– Month 13 onwards	0.00%	Beta	Alpha = 0 / Beta = 1375	
Treated AF to Disabling stroke– Month 1 to Month 12	0.01%	Beta	Alpha = 1 / Beta = 1374	
Treated AF to Disabling stroke– Month 13 onwards	0.00%	Beta	Alpha = 0 / Beta = 1375	
**Other clinical events rates for both arms (TAVI and SAVR) – Annual risk**				
Rehospitalisation – Month 13 onwards	0.0%	Gamma	SD = 0.00% (assumption)	SWEDEHEART registry (2018-2020)[6]
Reintervention rate -Year 1 onwards	0.1**%**	Gamma	SD = 0.01% (assumption)	
**Relative risk of death associated with treated AF., DS. and reintervention – for both arms**				
Treated AF	1.46	Lognormal	SD = 0.15 (assumption)	Odutayo et al.[28]
Disabling stroke	2.3	Lognormal	SD = 0.23 (assumption)	Dennis et al.[29]
Aortic reintervention	2.21	Lognormal	SD = 0.22 (assumption)	PARTNER 3 trial[9]<br>Procedural deaths (0.4%) compared to monthly mortality risk of general mortality for 73 years old[a] (0.16%)
**Disutilities associated to treated AF and disabling stroke – for both arms**				
Utility decrement: Treated AF	0.08	Gamma	SD = 0.00 (assumption)	Lanitis et al.[31]

Utility decrement: Disabling stroke	0.37	Gamma	SD = 0.04 (assumption)	
**Cost of the procedure (incl. rehabilitation)**				
TAVI with SAPIEN 3	302 329 SEK	Gamma	SD = 30232.92 (assumption)	Swedish Health Care Regions (2022) - Average of E03N across price lists for all regions
SAVR	244 750 SEK	Gamma	SD = 24474.99 (assumption)	Swedish Health Care Regions (2022) - Average of E04E across price lists for all regions
**Monthly cost associated to health states (Alive & well, Treated AF., DS.)**				
Treated AF up to 30 days	55 561 SEK	Gamma	SD = 5556.1 (assumption)	Hallberg et al.[30]
Treated AF – Month 2 onwards	2 111 SEK	Gamma	SD = 211.1 (assumption)	
Disabling stroke -up to 30 days	106 165 SEK	Gamma	SD =10616.5 (assumption)	Lanitis et al.[31]
Caregiver cost for disabling stroke -up to 30 days	69 491	Gamma	SD =6949.1 (assumption)	
Disabling stroke– Month 2 onwards	4 284 SEK	Gamma	SD = 428.4 (assumption)	
Caregiver cost for disabling stroke - Month 2 onwards	37 970 SEK	Gamma	SD = 3797.0 (assumption)	

Alive and well up to 1 year (both arms)	8 215 SEK	Gamma	SD = 821.5 (assumption)	Swedish Health Care Regions (2022) - Average of 'besök kardiolog' across price lists for all regions
Alive and well – Month 13 onwards (both arms)	8 215 SEK	Gamma	SD = 821.5 (assumption)	
**Other costs considered in the model – for both arms**				
Monthly cost of pacemaker complications	3 206 SEK	Gamma	SD = 320.57 (assumption)	Swedish Health Care Regions (2022) - Average of X31O across price lists for all regions
Rehospitalisation	61 340 SEK	Gamma	SD = 6133.9 (assumption)	Swedish Health Care Regions (2022) - Average of E47A, E47C and E47E across price lists for all regions

AF, atrial fibrillation; AKI, acute kidney injury; MI, myocardial Infarction; SAVR, surgical aortic valve replacement; TAVI, transcatheter aortic valve implantation; TIA, transient ischaemic attack.
